# Identification and characterisation of moderately thermostable diisobutyl phthalate degrading esterase from a Great Artesian Basin *Bacillus velezensis* NP05

**DOI:** 10.1016/j.btre.2024.e00840

**Published:** 2024-04-04

**Authors:** Brandon Mu, Pawel Sadowski, Junior Te'o, Bharat Patel, Nayana Pathiraja, Kevin Dudley

**Affiliations:** a Queensland University of Technology (QUT), The School of Biology and Environmental Science (BES), 2 George St Brisbane, QLD 4001, Australia; b Queensland University of Technology (QUT), Central Analytical Research Facility (CARF), 2 George St Brisbane, QLD 4001, Australia

**Keywords:** Diisobutyl phthalate, Enzymatic bioremediation, *Bacillus velezensis* Thermostable esterase, PAE's

## Abstract

Phthalate esters are known to be endocrine disrupting chemicals and are documented to pollute environments. Enzymatic degradation of PAEs is a potential bioremedial strategy to manage contamination. Thermostable bioremedial enzymes have advantages in enzyme manufacturing and storage. In this study, we identified, overexpressed, and characterised a moderately thermostable para-nitrobenzyl esterase from whole genome sequencing of a *Bacillus velezensis* NP05 from the Great Artesian Basin*,* capable of sequential 2-step hydrolysis of diisobutyl phthalate. The pnbA enzyme has a molecular weight of 55.14 kDa and pI of 5.31. It preferentially degrades para-nitrophenyl butanoate and has an optimal pH of 7–8. The pnbA esterase has an optimal temperature of 55 °C with a half-life of 4 h. Using HPLC we found that pnbA (0.122 U) can hydrolyse 0.83 mM of DIBP within 25 min. Lastly, pnbA is potentially a more economically viable candidate for enzymatic bioremediation of diisobutyl phthalate as a free enzyme.

## Highlights

• pnbA is moderately thermostable and potentially better suited for DIBP bioremediation as a free enzyme.
• Co-solvent effects were different between pNP-ester, and PAE substrates which other research groups should investigate.
• 83 % DIBP hydrolysis within 25 min with 0.122 U of pnbA at 60 °C and pH 8.
• pnbA is capable of PBAT plastic hydrolysis (see supplementary material).

## 1 Introduction

Phthalate esters (PAEs) are inexpensive and abundant plasticisers utilised in the polymer industry for a variety of products including packaging, flooring, paints, and cosmetics [1]. However, since the 1990′s researchers have raised concerns about the accumulation of PAEs in foods as a result of environmental contamination and leaching from plastic packaging [2], [3], [4], [5].

Phthalates are classified as endocrine-disrupting chemicals (EDCs) and have been associated with a variety of health conditions [6]. Phthalates are known to mimic endogenous endocrine molecules in humans and are therefore capable of triggering a variety of growth and development pathways, like the epidermal growth factor pathways and estrogen pathways [7]. They have been linked to a plethora of health issues such as phthalate syndrome, obesity, cardiovascular disease, dysgenesis syndrome, testicular cancer, hypospadias, endometriosis, androgenization, autism in boys, cryptorchidism, endocrine disruption in prenatal development, breast cancer, and uterine leiomyoma [2,3,5,[8], [9], [10], [11], [12], [13], [14], [15], [16], [17]].

Study by Trasande, Liu [18] have attributed to ∼ 90,000 premature deaths annually for Americans aged 55–64 by phthalate-related disorders resulting in societal costs of ∼40 billion dollars a year in economic productivity. Thus, there has been a great imperative to reduce the environmental contamination of PAEs.

Bioremediation is the process of managing organic contamination with the use of microorganisms or other biological means and has been suggested to be fast, cost-effective, and environmentally friendly [19]. There are numerous studies to date reporting various mesophilic enzymes involved in partial and complete degradation of PAE's [20], [21], [22], [23], [24]. However, there are limited studies on thermostable PAE degrading enzymes which are needed to understand and develop economically desirable enzymes for bioremediation [25,26]. Thermally robust PAE degrading enzymes are desirable as their inherent thermostability is beneficial in; enzyme production by heat-treatment purification from co-expressed mesophilic protein, medium to long-term stable storage capacity at room temperature, and long-term stable application in-situ bioremediation [27]. Finding thermostable PAE degrading enzymes can be difficult as microorganisms have had limited time to adapt to plastics and PAEs in the environment. So, a question arises. Where can such enzymes be found? Book chapter by Ogg, Spanevello [28], posit environments like the Great Artesian Basin (GAB) that have been in contact with hydrocarbon-containing materials could act as a source of thermostable bioremedial enzymes.

Unique to Australia is the Great Australian Artesian Basin. The GAB is intricately woven with hydrocarbon deposits and thermophiles are speculated to contain hydrocarbon-degrading thermozymes potentially useful in industries like biorecycling, bioremediation, and biofuels [28], [29], [30], [31]. In this work, we demonstrated GAB thermophiles contain potential bioremedial enzymes. In preliminary plastic degradation work, an enriched consortium containing *Anoxybacillus* sp. and *Bacillus* sp. was observed to biodeteriorate Nylon 6 films according to metabolomics, and scanning electron microscopy (SEM), under synthetic media conditions at 55 °C (see supplementary material). We believe thermophiles from the GAB contain thermostable enzymes for PAE bioremediation. Diisobutyl phthalate (DIBP) is a widely used phthalate found in the environment and has a well-established negative impact on human health [32,33]. In this study, we describe a moderately thermostable para-nitrobenzyl esterase (pnbA) hydrolytic enzyme from a moderate thermophile *Bacillus velezensis* NP05, isolated from the GAB. The enzyme was found capable of two-step hydrolysis of DIBP to mono-isobutyl phthalate (MIBP) and phthalic acid (PTH) with a broad enzymatic activity range pH range, moderate thermostability and preliminary evidence of polybutylene adipate terephthalate plastic (PBAT) hydrolysis.

## 2 Materials and methods

### 2.1 Strains and reagents

*Escherichia coli* Tuner (DE3) strains were purchased from Novagen (Merck). DIBP, MIBP, and PTH at 99 % purity were purchased from Merck. HisPur™ Ni-NTA Spin columns (3 mL), were purchased from Thermo Fischer Scientific™. P-nitrophenyl-octanoate (pNP-C8), pNP-hexanoate (pNP-C6), pNP-butyrate (pNP-C4), and pNP-acetate (pNP-C2) esters were purchased from TCI and Merck. All other chemicals were HPLC-grade.

### 2.2 Whole genome sequencing and analysis

An attempt was made to purify and sequence *Bacillus* sp. genomic DNA for whole genome sequencing from an enriched culture containing *Anoxybacillus* sp. and *Bacillus* sp. observed to biodeteriorate Nylon 6. Genomic DNA from cells grown in 0.2 % tryptone soyabroth at 45 °C, at 200 rpm for 2–3 days were collected by a MagAttract Power Microbiome DNA/RNA kit (QIAGEN) following the manufacturer's protocol. Library preparation and sequencing were performed at the Central Analytical Research Facility (CARF) at Queensland University of Technology (QUT). The library was sequenced with a PacBio Sequel system sequencer on 2 SMRT Cells with 10-hour movie times. The raw bam files were deconvoluted by barcodes using the PacBio lima tool. The barcoded subread BAM files were then converted to FASTA format via SAMtools. Fasta files were then concatenated, and d*e novo* assembly was performed in Flye V.2.9.2 (Flye, RRID: SCR_017016) [34], for PacBio data with default parameters on 4 threads. The assembled genome was then checked for completeness using Benchmarking Universal Single-Copy Orthologs (BUSCO) V5.4.6 [35], using the “bacillales_odb10” lineage dataset. Broad taxonomic assessment of the draft genome was checked using Genome Taxonomy Database Tool Kit (GTDB-TK) V2.3.2 [36].

The *B. velezensis* genome was annotated using the rapid prokaryotic genome annotation tool Prokka [37]. The enzyme pnbA was selected based on the protein Basic Local Alignment Search Tool (BLAST) results of the annotated proteome to a compiled list of plastic degrading enzymes pulled from the Plastic Microbial Degradation Database (PMBD) (accessed 02/10/2020) (http://pmbd.genome-mining.cn/home) [38] (see supplementary material). The putative functions were inferred based on Pfam domain analysis (http://pfam-legacy.xfam.org/) and protein BLAST. Multiple sequence alignment (Clustal Omega default settings) in JalView (Version 2.11.2.7) and ESPript (https://espript.ibcp.fr) was utilised to align and visualise alignment, respectively. The signal sequence for peptide cleavage in pnbA was predicted by signal-P 5.0 (https://services.healthtech.dtu.dk/services/SignalP-5.0/). The mass and isoelectric point of pnbA was predicted utilizing the pI/MW tool through Expasy (https://www.expasy.org/). EMBOSS's needle webserver was utilized for pairwise alignments (https://www.ebi.ac.uk/jdispatcher/psa/emboss_needle).

### 2.3 Modelling and molecular docking

AlphaFold 2.0 is a template-based method to predict protein structure that won CASP 14 in 2020. Chimera X daily development build 1.4.dev 202204260114 (2022-04-26) was downloaded and used to predict, model, and annotate pnbA. pnbA's peptide sequence was imported into Chimera X, and the structure was constructed with the incorporated AlphaFold 2.0 program. USCF chimera version 1.16 was downloaded to dock and simulate protein-substrate interactions. DIBP was drawn in the online chemical drawing tool MolView (https://molview.org/) and exported into PDB format. The predicted AlphaFold pnbA structure and DIBP structure was loaded into UCSF chimera. An AutoDock Vina search volume was then drawn within the active site of pnbA around Serine-190. DIBP substrate was docked to the active site of pnbA through AutoDock Vina. The enzyme docked with DIBP was exported to PDB file format and uploaded into Maestro v13.9 to display ligand–protein interactions.

### 2.4 Expression and purification

The pnbA gene was modified to incorporate a polyhistidine-tag in-frame of the N-terminus of the enzyme and the nucleotide sequence was codon optimised for *E. coli* hosts. The optimised sequence was then synthesised and cloned into a pET-Duet-1 expression vector through GenScript's molecular biology service. The vector was transformed into *E. coli* Tuner (DE3) cells as per the manufacturer's protocol. Protein purification was accomplished by growing and inducing 50 mL of transformant cells as follows: 50 mL of Luria-Bertani (LB) broth containing 100µg·mL^−1^ ampicillin was inoculated from a single colony of the transformant grown on an LB agar plate with 100 µg·mL^−1^ ampicillin. Seed culture was grown overnight at 37 °C at 250 rpm. 2.5 mL of seed culture with an OD_600_ of ≥ 4 was used to inoculate 50 mL of Terrific Broth (TB) with 100 µg mL^−1^ ampicillin. The culture was then grown at 37 °C, at 250 rpm until OD_600_ reached a value of one. Expression was then induced with Isopropyl β-d-1-thiogalactopyranoside (IPTG) at a final concentration of 0.5 mM and grown for an additional 3 h. Cells were then harvested by centrifugation at 5000 rpm for 15 min in an Allegra X-15R centrifuge. The supernatant was then discarded, and cells were then resuspended in 50 mL TES buffer (50 mM Tris-HLC, 5 mM EDTA, 50 mM NaCl, pH 7.5). Cell lysates were then extracted via cell homogenization using the continuous flow cell disruptor CF1 (Constant Systems Ltd.) by passing 500 ml of 80/20 ethanol-water solution and then three subsequent passes with DI water at 40,000 PSI. Cell suspensions were then passed through the cell disruptor three times at 27,000 PSI. Purification of the lysate was then carried out as per the protocol of HisPur™ Ni-NTA Spin Column 3 mL kit, and dialysed via SnakeSkin™ Dialysis Tubing, 10 K Molecular weight cut off (MWCO), 22 mm with Phosphate buffer (20 mM NaH_2_PO_4_, 0.3 M NaCl, pH 7.4). Buffer was exchanged a total of three times with 2 h per exchange at room temperature. The success of the expression and purification was examined using Image J analysis of sodium dodecyl sulphate-polyacrylamide gel electrophoresis (SDS-PAGE), and the protein concentration was estimated by bicinchoninic acid assay using bovine serum albumin (BSA) as standards (Peirce^tm^).

### 2.5 Enzymatic activity

Enzyme activity was determined using a Varioskan LUX plate reader based on the release of p-nitrophenol from p-nitrophenyl (pNP) ester by the enzyme monitored at 410 nm in triplicate over a four-minute assay. The standard assay was measured at 45 °C with 1 mM pNP-ester substrate in 60 mM Universal Buffer (UB) (Britton-Robinson buffer) (pH 8) and 115.344 ng (0.57572 µg/mL final concentration) of purified pnbA in 200 uL total volume, unless otherwise specified. The pNP-butyrate substrate was used in standard conditions unless otherwise specified. Blank pNP-ester-only controls were measured alongside experimental groups to subtract appropriate values for non-enzymatic hydrolysis of the pNP-substrate. One unit of enzyme activity (U) was defined as the amount of activity required to release 1 µmol of p-nitrophenol from the pNP-ester standard under assay conditions. The relative activity of pnbA on pNP-ester substrates (pNP-C2, pNP-C4, pNP-C6, and pNP-C8) was determined during the non-limiting linear range four-minute reactions and performed in triplicate. The pNP-esters were first dissolved in HPLC-grade acetonitrile to make stock solutions at 10 mM.

For phthalate degradation characterization, DIBP was dissolved in HPLC-grade methanol first at a concentration of 20 mM. DIBP enzymatic degradation was performed in 60 mM UB buffer (pH 8) and at 60 °C to mimic thermophilic conditions. DIBP was loaded to a final concentration of 1 mM and reacted for 30 min with the enzyme at a final concentration of (1.503752 µg/mL (0.122 U)) in GCMS vials with 5 % v/v methanol co-solvent in 1 mL total volume. The amount of DIBP, MIBP, and PTH was then determined by HPLC every 5 min. Three independent determinations were performed for every time point. Substrate-free enzyme assays were used as blank negative controls, and enzyme-free assays with substrate were used as control groups. Microsoft Excel was used to plot data and conduct statistical analysis.

### 2.6 High-performance liquid chromatography

High performance liquid chromatography (HPLC) methods relied on a study by Ding, Wang [20], with the following changes: Reaction mixture (1 mL) of enzyme and DIBP in 60 mM UB buffer (pH 8) was incubated at 60 °C for up to 30 min. After set-time incubation, the reactions were terminated using 100 µL of 1 M HCl. Organic compounds were extracted by agitation using 700 µL ethyl acetate, and 500 µL of the ethyl acetate phase was collected and dried in a vacuum concentrator with heating (54 °C at 63 mbar). Samples were reconstituted in 500 mL of HPLC-grade methanol and analysed using an Agilent 1100 Series HPLC (Agilent) equipped with a diode array detector, monitored at a wavelength of 360 nm. The mobile phase consisted of an 85:15 (v/v) pre-mix of 0.1 % phosphorus acid in water and acetonitrile that was running at 1 mL · min^−1^ through an Eclipse XBD-C18 (5 µm, 4.6 x 150 mm) column with guard (XBD-C18 cartridge) and maintained at 30 °C. Quantification involved generating standard curves of DIBP, MIBP, and PTH in the range of 0–2 mM.

### 2.7 Biochemical characterization

Biochemical characterization of pnbA was examined by standard pNP-ester assay with different temperatures, pH's, organic co-solvents, chemical reagents, and metal ions. The effect on pH was tested at 45 °C by adjusting the Universal Buffer's pH (4–10) and measured at 348 nm, p-nitrophenol's pH-independent isosbestic wavelength [39]. The effect of temperature was tested between the temperatures of 40 °C to 80 °C, at a pH of 8, and using pNP-C8 as a thermostable substrate. The thermostability of pnbA was determined by standard assay at 45 °C, an assay time of 90 s, at a pH of 8, and pNP-C4 as the substrate, after pre-incubating pnbA at the temperatures of 40 °C to 80 °C for 7 h with a sample taken every hour and centrifuged at 4 °C at 5000 rpm. The activity of a zero-hour heat-treated sample was regarded to have 100 % activity. The thermodynamic parameters of deactivation rate constant (of enzyme) (K_d_), half-life (t_1/2_, hours), enthalpy (H), entropy (S), and Gibbs free energy (G) were derived using the Arrhenius, and Ehring equations based on methods by Singh and Chhatpar [40]. The effect of inhibitors or activators (metal ions, organic co-solvents, and chemical reagents) on pnbA's activity was determined by standard pNP-C4 assay with a pH, and temperature of 8, and 45 °C respectively. The activity was measured after 4 min of incubation. The assay of pnbA without metal ions, organic co-solvents, and chemical reagents was defined as the control. The effects of organic co-solvents were also tested on pnbA activity on DIBP.

## 3 Results and discussion

### 3.1 Whole genome sequencing, sequence analysis, and identification

For whole genome sequencing, two SMRT cell chips were sequenced producing two ten-hour movies. From the Flye assembly, there was a total read length of 7,824,758 bp generating four contigs with 11,176 N50 subreads with a median length of 4,007,403 bp, a mean coverage of 55, with 0 scaffolds. Two genomes were fully resolved from the assembly and the *Bacillus* sp. genome had an estimated length of 4,007,403 bp with ∼ 52x coverage and 4011 genes. From the BUSCO analysis, the draft genome was 98 % complete with a total of 450 BUSCO's searched with 441 being complete and single copy, one missing, eight fragmented, and zero duplicated. The draft genome was identified to be a *Bacillus velezensis* through GTDB-TK with an Average Nucleotide Identity (ANI) of 98.08 % to *B. velezensis* (GCF_001461825.1) type-strain. Raw sequence data can be found at the Sequence Read Archive (SRA) under the project name: PRJNA1044461 (SRR27003626).

**Fig. 1:**
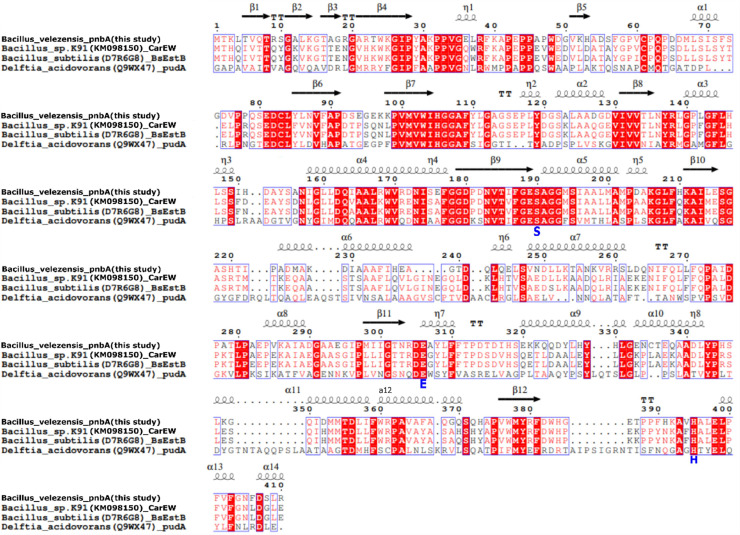
Amino acid sequence alignment (ES pript) of Bacillus velezensis NP05 pnbA and its homologs. The protein accession number (GenBank) and protein name are as follows: pnbA (OR468330), CarEW (KM098150) from Bacillus sp. K91, BsEstB (D7R6G8) from Bacillus subtilis, PudA (Q9WX47) from Delfita acidovorans TB-35. The catalytic triad (Ser190(S), Glu306(E), and His395(H)) is shown underneath by blue characters. Pentapeptide (Gly-X-Ser-X-Gly) can be seen at positions 188–192. Arrows represent β sheets, and loops represent α helixes. Red boxes and white letters indicate fully conserved residues, while red letters represent residues with identical physiochemical properties. TT represents α and β corners.

Blasting the Prokka-annotated *B. velezensis* NP05 proteome to the PMBD database revealed a para-nitrobenzyl esterase gene with a 1449-bp open reading frame encoding a 483 amino acid protein without a typical bacterial signal sequence motif according to signal-P 5.0 [41]. The pnbA gene was found to have 63 % sequence similarity at the amino acid level to CarEW, a known mesophilic DIBP hydrolysing enzyme [20]. Furthermore, pnbA was found to be 100 % similar in nucleotide sequence identity *to B. velezensis* strain UA2208 carboxylesterases (accession no. CP097586.1) and 99 % nucleotide sequence identity to *B. velezensis* strains HN-Q-8 (accession no. CP045711.1), and SRCM116265 (accession no. CP103856.1). The pnbA carboxylesterase was found to be ubiquitous in all *B. velezensis* strains [42], [43], [44]. Multiple sequence alignment (MSA) of pnbA (GenBank accession no. OR468330), CarEW (GenBank accession no. KM098150) from *Bacillus* sp. K91 Ding, Wang [20], BsEstB (GenBank accession no. D7R6G8) from *Bacillus subtilis* Ribitsch, Heumann [45] and PudA (GenBank accession no. Q9WX47) from *Delfita acidovorans TB-35* Akutsu, Nakajima-Kambe [46], Nomura, Shigeno-Akutsu [47] (Fig. 1) shows pnbA is a carboxylesterase containing a catalytic triad of Ser190-Glu306-His395 and the consensus pentapeptide motif (Gly-X-Ser-X-Gly) around the active site. The theoretical molecular weight (MW) and isoelectric point (pI) of pnbA were calculated as 55.14 and 5.31 respectively [48]. To our knowledge, *B. velezensis* pnbA activity on DIBP, pNP-ester substrates, or PBAT polymer has not been characterised to date. Nor has its thermostability been established.

### 3.2 Expression, purification, and substrate specificity analysis

**Fig. 2:**
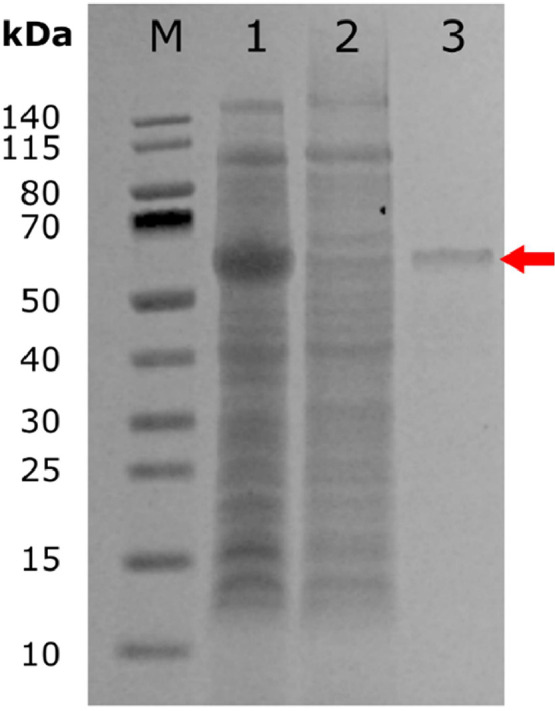
SDS-PAGE of *E. coli* Tuner (DE3)-expressed pnbA. 4–12 % Bis-Tris NuPage gel was stained using AquaStain. From the left: (Lane M) PageRuler^tm^ Prestained Protein Ladder, 10 to 140 kDa. (Lane 1) *E. coli* crude cell lysate induced with IPTG (1.994 g/L). (Lane 2) cell lysate flowthrough with pnbA removed. (Lane 3) *E. coli* crude cell lysate induced with IPTG and additionally passed through an Ni-NTA metal affinity column, where the pnbA band can be seen at ∼55.14 kDa (red arrow, 28.836 mg/L).

To ascertain the activity of pnbA, we have expressed it as a His-tagged fusion protein in a pETDuet-1 vector, induced with isopropyl Isopropyl β-d-1-thiogalactopyranoside (IPTG), and purified on a nickel nitrilotriacetic acid (Ni-NTA) columns. SDS-PAGE (Fig. 2. Lane 1) shows pnbA expression with background proteins totalling 1.994 g/L. In (Fig. 2. Lane 2) the band was absent in flow through crude cell lysate with pnbA removed. In (Fig. 2. Lane 3) shows a single band corresponding to the pnbA fusion protein (red arrow) with a theoretically predicted mass of ∼55.14 kDa, a yield of 28.836 mg/L with a purity of 48 %, totalling a removal of 98.5 % of protein. Substrate specificity analysis revealed that at 45 °C and pH 8 the enzyme activity of pnbA was highest towards pNP-C4 followed by pNP-C6, DIBP, pNP-C8 and pNP-C2 as seen in (Table 1).

**Table 1:** The activity of pnbA towards pNP-ester substrates.

Substrate	Enzyme units (µmol min^−1^)	Enzyme activity (µmol · min^−1^ · mL^−1^)	Specific activity (µmol · min^−1^ · mg^−1^)
pNP-C2	1.69 × 10^−3^	0.17	14.69
pNP-C4	16.05 × 10^−3^	1.607	139.19
pNP-C6	6.41 × 10^−3^	0.647	55.58
pNP-C8	2.27 × 10^−3^	0.23	19.67

### 3.3 Effect of temperature and pH

**Fig. 3:**
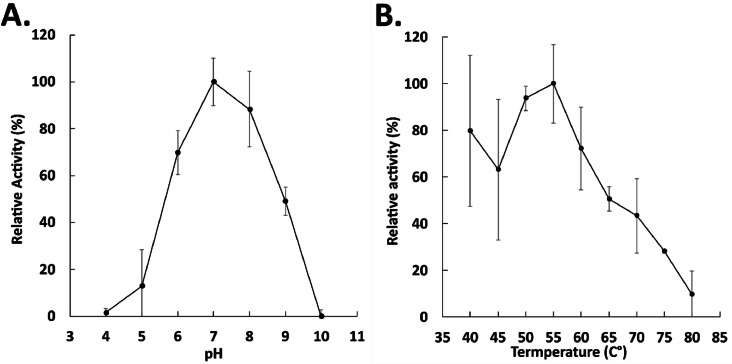
Effect of pH (A.) and temperature (B.) on pnbA activity towards pNP-C4 and pNP-C8 respectively. Error bars represent 95 % C.I. *n* = 3.

The effect of pH was determined using pNP-C4 as the standard substrate at 45 °C, measuring at 348 nm with pH values ranging from 4 to 10 (Fig. 3, A). The maximal activity of pnbA was observed at a pH of 7–8. Changes in pH to < 5 or > 9 were associated with an activity drop of ≤ 20 %. The pH-associated activity drop was attributed to non-reversible denaturation [49].

**Fig. 4:**
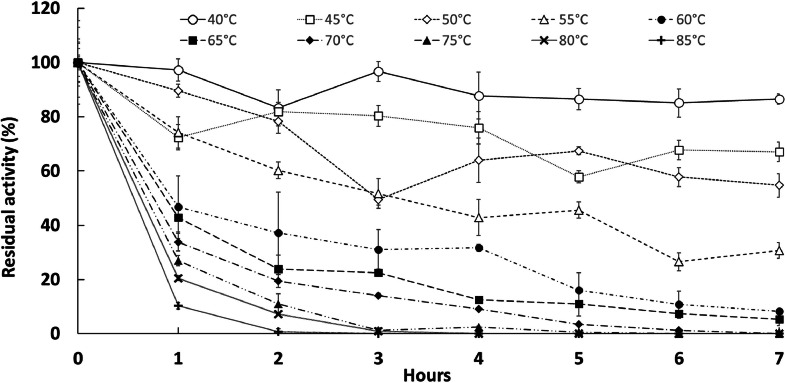
Thermostability of pnbA at different temperatures measured by residual activity by standard assay. Error bars represent 95 % C.I. *n* = 3.

**Fig. 5:**
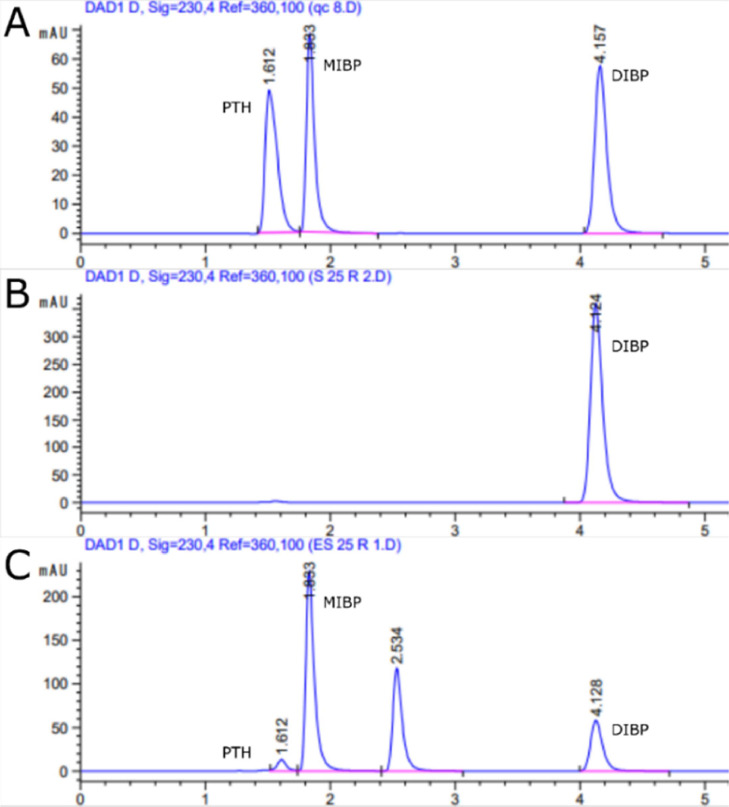
HPLC analysis (target wavelength 230 nm, background correction wavelength 320 nm) of DIBP, MIBP, and PTH. (A.) QC sample with DIBP, MIBP, and PTH (0.5 mM). (B.) DIBP after 25 min of incubation without pnbA. (C.) DIBP after 25-minute incubation with pnbA.

The effect of temperature was investigated in the range of 40–80 °C using pNP-C8 as the standard substrate at pH 8 measuring at 410 nm (Fig. 3, B). Activity was found to be increasing up to 55 °C and then dropped at higher temperatures reducing to 9.5 % activity at 80 °C. In the range of 40–60 °C, pnbA displays activities of ≥60 % relative activity and achieves optimal activity at 55 °C (Fig. 3, B). Accordingly, the pnbA enzyme was considered a moderately thermophilic esterase. The thermostability of pnbA was measured by pre-incubating the purified enzyme for 7 h at temperatures ranging from 40 to 80 °C with hourly sampling (Fig. 4). Protein aggregates were removed using centrifugation and clear supernatant aliquots were subjected to a standard assay with pNP-C4 as the substrate, at pH 8 and 45 °C. The pnbA enzyme retained ≥60 % of its activity after 7 h of pre-incubation at 40, 45 and 50 °C. At the optimal temperature of 55 °C, pnbA retained 30 % activity (Fig. 5). At 40, 45, 50 and 55 °C, pnbA had corresponding half-lives of 34.83, 13.75, 8.81 and 4.03 h (Table 2). The pnbA's moderate thermostability may prove to be a beneficial attribute in *in-situ* plasticiser degradation applications.

**Table 2:** Half-life (t_1/2_), deactivation rate constants (K_d_) and thermodynamic parameters enthalpy (ΔH_d_), entropy (ΔS_d_), and Gibbs free energy of denaturation (ΔG_d_) of pnbA at different temperatures.

Temperature ( °C)	t_1/2_ (Hours)	K_d_	ΔH_d_ (J/mol)	ΔS_d_ (J/mol)	ΔG_d_ (J/mol)
**40** °**C**	**34.83**	**0.02**	**93,708.09**	**19.02**	**87,750.99**
**45** °**C**	**13.75**	**0.05**	**92,788.19**	**21.95**	**85,804.39**
**50** °**C**	**8.81**	**0.08**	**93,091.01**	**21.00**	**86,303.21**
**55** °**C**	**4.03**	**0.17**	**92,441.78**	**22.99**	**84,897.93**
**60** °**C**	**2.13**	**0.33**	**92,123.81**	**23.99**	**84,155.19**
**65** °**C**	**1.80**	**0.39**	**93,071.95**	**21.08**	**85,944.66**
**70** °**C**	**1.05**	**0.66**	**92,957.17**	**21.42**	**85,608.27**
**75** °**C**	**0.67**	**1.03**	**93,065.92**	**21.10**	**85,718.96**
**80** °**C**	**0.45**	**1.56**	**93,233.61**	**20.63**	**85,947.53**
**85** °**C**	**0.28**	**2.44**	**93,243.71**	**20.61**	**85,860.92**

Thermodynamic parameters ΔH_d_, ΔS_d,_ and ΔG_d_ suggest the reaction is non-spontaneous and requires energy for the reaction to occur (Table 2). Enthalpies and entropies are positive. The positive entropy values signify an increase in disorder after the protein's thermal deactivation to the denatured state, and the positive enthalpies suggest an endothermic reaction, requiring free energy for the reaction to occur. These values suggest denaturation reaction is temperature-dependent with low reactivity at low temperatures and higher reactivity at higher temperatures [50]. This is reflected in the half-lives as the higher temperature shortens the half-life as there is more abundant energy to denature the protein.

### 3.4 Effect of additives

The effect of different metal ions and chemical reagents on pnbA activity was performed at pH 8, on pNP-C4 substrate, and at 60 °C to replicate thermophilic conditions. According to the results in (Table 3), no co-factors or chemical reagents improved activity with the potential exception of magnesium. Calcium had no apparent effect on activity while Cobalt, aluminium, potassium, Manganese, and Sodium, had weak inhibitory effects. Zinc, and Copper, had a moderate inhibitory effect. Chemical reagent Urea had a weak inhibitory effect, while DTT and Triton X-100 had moderate inhibition. Lastly, Tween 80 and SDS had strong inhibition.

**Table 3:** Effect of chemical reagent and metal ion additives on pnbA activity on pNP-C4 hydrolysis.

Metal ions/Chemical reagents	Concentration	Relative activity (%)
Control	**1 mM**	**100 ± 8.3**
Mg^2+^	**1 mM**	**110 ± 5.9**
Ca^2+^	**1 mM**	**101 ± 8.3**
Co^2+^	**1 mM**	**98 ± 8.8**
Al^2+^	**1 mM**	**96 ± 1.6**
*K*^+^	**1 mM**	**92 ± 5.2**
Mn^2+^	**1 mM**	**88 ± 13.3**
Urea	**1 mM**	**87 ± 5.2**
Na^+^	**1 mM**	**87 ± 6.6**
Zn^2+^	**1 mM**	**44 ± 3.6**
Cu^2+^	**1 mM**	**33 ± 4.3**
DTT	**2 %**	**74 ± 12.6**
Triton X-100	**2 %**	**33 ± 4.8**
Tween 80	**2 %**	**16 ± 2.3**
SDS	**2 %**	**3±2.0**

The effect of organic cosolvents on enzyme activity is important for industrial applications and was tested under a variety of different co-solvents by making pNP-C4 stocks in Acetonitrile, Methanol, Ethanol, Isopropanol, Acetone, and DMSO. Co-solvents made up a concentration of 5 % v/v under standard assay conditions (pH 8, 60 °C, pNP-C4). Table 4 shows Methanol, Ethanol, Isopropanol, and Acetone weakly inhibited pnbA activity (76 %, 94 %, 92 %, and 82 % respectively), while DMSO moderately inhibited activity (64 %) on pNP-C4 hydrolysis compared to the control. The cosolvents tested here are used widely in the plastic industry and are likely to co-contaminate the environment with PAEs. The stability of pnbA in the presence of these organic contaminants is critical in the efficacy of PAE degradation. Lastly, the effects of cosolvents were also examined on pnbA's activity on DIBP. Table 5 shows Methanol, Acetone, Ethanol, and Isopropanol strongly activated (322 %, 220 %, 256 % and 340 % respectively) pnbA activity on DIBP hydrolysis compared to the control. This is interesting as there is an obvious discrepancy of cosolvent effects on pnbA activity which is dependent on the substrate being degraded. One possible explanation is cosolvents influence the overall solution polarity, and this may favour products, pushing the enzymatic reaction mechanism forward, or favour substrates and hold back the reaction [51]. DMSO was not tested as it was not available at the time. According to these results, it would be interesting if other research groups tested co-solvent effects on pNP-ester substrates in parallel with PAE substrates to see if inhibition or activation is substrate dependant. The effects of metal ion and chemical reagents of DIBP enzymatic hydrolysis were not investigated in this study but may also yield different activities depending on the substrate.

**Table 4:** Effect of co-solvent on pnbA activity on pNP-C4 hydrolysis.

Organic Co-solvent	Concentration (v/v)	Residual activity (%)
Acetonitrile (Control)	**5 %**	**100 ± 12.7**
Methanol	**5 %**	**76.5±6.0**
Ethanol	**5 %**	**94.7±6.7**
Acetone	**5 %**	**82.2±4.8**
Isopropanol	**5 %**	**92.3±8.5**
DMSO	**5 %**	**64.2±15.6**

**Table 5:** Effect of co-solvent on pnbA activity on DIBP hydrolysis.

Organic Co-solvent	Concentration (v/v)	Relative activity (%)
Acetonitrile (Control)	**5 %**	**100± 9.6**
Methanol	**5 %**	**322.3± 42.9**
Ethanol	**5 %**	**256.9± 39.9**
Acetone	**5 %**	**220.2± 42.6**
Isopropanol	**5 %**	**340.6± 36.1**

### 3.5 DIBP enzymatic degradation

**Fig. 6:**
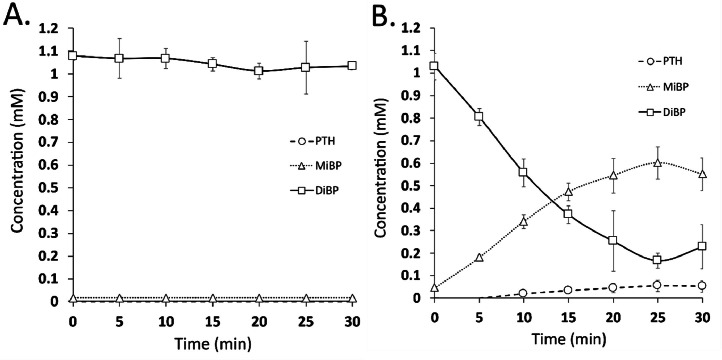
DIBP enzymatic hydrolysis, (A.) without pnbA, (B.) with pnbA. Error bars are 95 % C.I, *n* = 3.

**Fig. 7:**
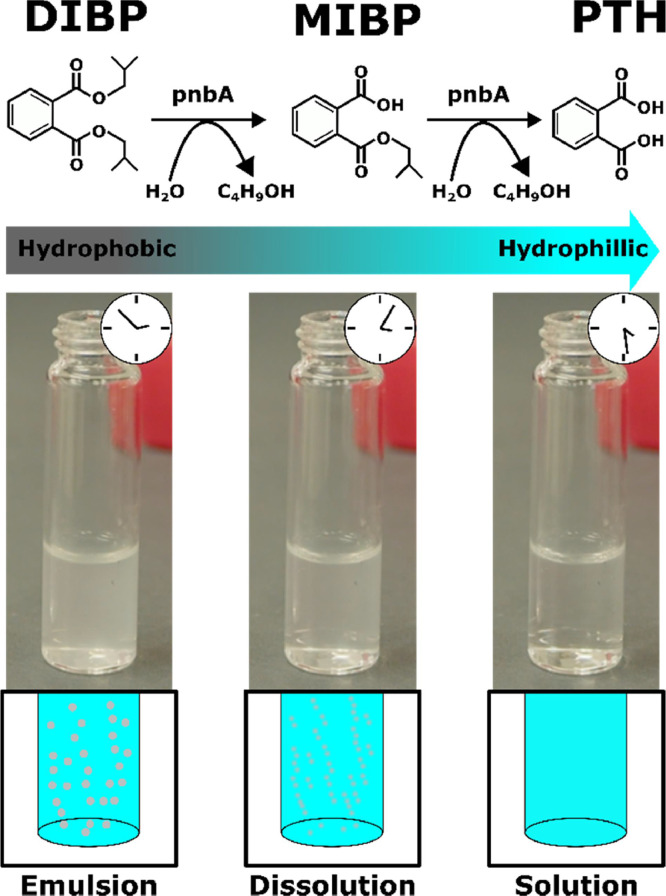
DIBP enzymatic degradation to polar hydrolysis products MIBP and PTH. Conversion of DIBP ester to more polar MIBP, and PTH carboxylic acids turning the emulsion into a homogenous solution as the reaction proceeds.

The hydrolysing capabilities of pnbA on DIBP were determined by HPLC quantitation of DIBP and its hydrolysis products MIBP, and PTH. The retention times of DIBP, MIBP, and PTH were 4.12, 1.83, and 1.60 min respectively in the quality controls (QC's), standards, and samples (Fig. 5, A). No hydrolysis of DIBP was seen in DIBP reactions without enzyme (Fig. 5, B). DIBP, MIBP and PTH were identified in reactions of DIBP with enzyme (at 60 °C after 25 min) (Fig. 5. C).

(Fig. 6) shows DIBP hydrolysis in the absence of enzyme (Fig. 6, A) and in the presence of enzyme (Fig. 6, B). As can be seen without pnbA, DIBP is not hydrolysed. While the addition of pnbA hydrolyses DIBP to MIBP and PTH. There is a stoichiometric consistency (∼ 1 mM) in DIBP, MIBP, and PTH concentrations across the sampling periods. (Fig. 7) shows DIBP creates an emulsion, which clears after enzymatic hydrolysis of DIBP to its more polar products. The degradation of DIBP is suggested to be stepwise hydrolysis and the degradation products MIBP and PTH are also reported in a study by Ding, Wang [20]. In total, we found that 0.122 U of pnbA (calculated from pNP-C4 enzyme units) can hydrolyse 0.83 mM of DIBP within 25 min. In (Fig. 5, C) the chromatogram shows an unidentified peak at 2.534 min which was present only in experimental samples but not in any of the control groups. It is suspected that the peak is a trans-esterified by-product “isobutyl methyl phthalate” as there is methanol present as a co-solvent which may have intermediate polarity between DIBP, and MIBP. In the chromatograms of experimental samples with acetonitrile co-solvent (see supplementary material) the unidentified peak is not seen. Simultaneously, in chromatograms of experimental samples with ethanol co-solvent (see supplementary material) there are different unidentified peaks. The unidentified peaks would interfere with the stoichiometric relationship of DIBP, MIBP, and PTH. Thus, the unidentified peak is more likely an introduced contaminant rather than a side product. Determination of the unidentified peaks is beyond the scope of this study but could be resolved through liquid chromatography mass spectrometry in later studies.

### 3.6 Phthalate esterase comparison and molecular docking analysis

The pnbA enzyme is a non-novel enzyme and has been reported in other *B. velezensis* genomes as a core gene but its activity on standard pNP-ester or DIBP has not been well studied. Although not novel it should not be overlooked as it has demonstrated novel phthalate and plastic degrading capabilities. There are a handful of studies of *Bacillus* genera phthalate degrading esterases [20,23,24,52]. A study by Ding, Wang [20] reported a CarEW capable of two-step hydrolysis of DIBP to MIBP and PTH. This studies pnbA has ∼63 % amino acid sequence identity to CarEW based on EMBOSS needle pairwise analysis (see supplementary material). Compared to CarEW (Table 6), pnbA appears more thermostable and therefore, potentially a better candidate for DIBP hydrolysis in bioremediation applications. This could be due to pnbA having more alanine and glycine residues (ALA = 54, GLY = 37 VS ALA = 53, GLY = 33) than CarEW which are associated with thermostable proteins [27]. Additionally, CarEW has a broader pH range (6.5–9.5) than pnbA (6–8). The presence of more thermostabilizing residues may conversely narrow the pH optimum of an enzyme [53]. It is suggested other research groups consider this as a potential tradeoff. Unfortunately, a direct comparison of DIBP degradation cannot be made, as Michalis-menten kinetics were not performed in this study but can be explored in future research. So it is not known which enzyme is better at hydrolyzing DIBP. Lastly, to briefly mention, a follow-up study by Ding, Zhou [54] research group found CarEW had improved thermostability as a fusion protein with ice nucleation protein (inpn) which improved activity and thermostability. This strategy could also be employed to improve the activity and thermostability of pnbA in a future study.

**Table 6:** Comparison of residual activity (%) after 1-hour heat treatment to previously characterised DIBP degrading bacterial esterase.

Temperature	pnbA (OR468330)	CarEW [20]
45 °C	**90 %**	**60 %**
55 °C	**75 %**	**40 %**

**Fig. 8:**
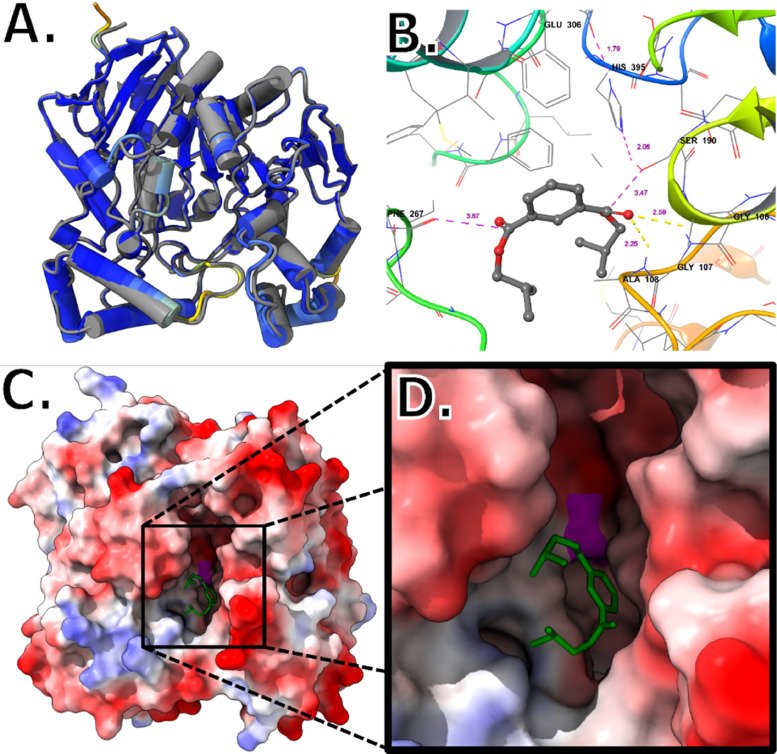
In-silico bioinformatic analysis of pnbA and DIBP. (A.) overlay of AlphaFold 2 structure of this studies pnbA (blue-yellow) on top of crystal structure pnbA (grey) from Bacillus subtilis 168 (P37967). (B.) Molecular docking of DIBP in pnbA with inter-atomic distances angstrom (Å). (C.) Electrostatic potential of pnbA (red = negative, blue positive) with DIBP docked in the catalytic pocket. (D.) Close-up of pnbA catalytic pocket with DIBP docked (Serine-190 surface highlighted in magenta).

EMBOSS needle pairwise analysis of *Bacillus* sp. LUNF1 (dphBL1) [52], to this studies pnbA shows a 99.6 % similarity based on the sequence alignment. A total of 8 amino acids are different at various positions in comparison of pnbA to dphBL1 (see supplementary material). For pnbA, at position 78, hydrophobic alanine is substituted by proline. At position, 198 polar serine is substituted by hydrophobic alanine. Lastly, at positions 256, and 421 polar asparagine's are substituted by charged aspartic acids [55]. This leaves pnbA overall, slightly more hydrophilically polar (uncharged) than near identical dphBL1. The differences in these sequence positions do not adversely affect the tertiary structure between the two. It can be seen the predicted AlphaFold structure of pnbA is identical to the crystal structure of pnbA from *Bacillus subtilis* 168 (Fig. 8A.). The study by Fan, Li [52] research group showed similar results in modelling. The dphBL1 esterase is capable of degrading Dibutyl phthalate (DBP), Diethyl phthalate (DEP), and Benzyl butyl phthalate (BBP). However, they only report hydrolysis to mono-form phthalate and not complete hydrolysis to PTH. The pnbA esterase from this study seems to be able to degrade more DIBP (83 %) than dphBL1 can degrade DBP (64.7 %), DEP (65.0 %), or BBP (46.2 %) under ideal conditions from a rudimentary perspective [52]. The dphBL1 esterase exhibits broader pH activity from 6 to 9 and an optimum temperature of 40 °C. They did not ascertain the thermostability of dphBL1, so it is not known if dphBL1 is thermostable. However, given the optimum activity for pnbA in this study is 55 °C and the dphBL1 esterase is 40 °C [52], it is tenable to say pnbA has a higher thermostability. This is weakly supported by the fact that pnbA has one more alanine residue than dphBL1. (Fig. 8. C., and D.) shows the DIBP docked into pnbA with a docking affinity of −5.6. It can be observed the DIBP is not conformationally strained to fit in the catalytic site. The inter-atomic distances in (Fig. 8. B.) suggest that hydrolysis is tenable as the catalytic Serine-190 is only 3.47 Å away from the DIBP carbonyl ester and is within range for nucleophilic attack. Molecular docking simulation of dpHBL1 to DIBP substrate reveals a docking score of −6.2 (see supplementary material) but is not close to the catalytic serine. The 8 amino acid residue difference does not seem to interfere with the binding of DIBP to dphBL1. However, (pose 8) shows molecular docking simulations of DIBP that puts carbonyl near catalytic serine and has a weaker affinity of -5.6 which may suggest that dphBL1 could also hydrolyse DIBP, and conversely that pnbA could degrade BBP, DMP, and DBP.

The study by Fan, Li [52] also performed site-directed mutagenesis in an attempt to produce a variety of dpbBL1 mutants to understand important residues in DBP hydrolysis. Although no mutants yielded higher activity than the wild-type dphBL1, they mention the importance of π-π interactions of Phenylanaline-310 to DBP and mention the importance of a variety of other residues. Unfortunately, site-directed mutagenesis was beyond the scope of this study, but site-directed mutagenesis of Phenylanaline-310 in pnbA would be a good starting point in future research to better understand the hydrolysis of DIBP as it contains a benzene group as well. Moreover, site-mutagenesis of specific residues to alanine residue may be an interesting method of improving thermostability or broaden the effective temperature range, improving the pnbA's aspects for economic production [28]. To briefly mention, molecular docking analysis of Phenylanaline-267, Alanine 108, and Glycine 107 (Fig. 8. B.) contribute to holding the DIBP substrate in the catalytic pocket and are potential sites for site-directed mutagenesis. Furthermore, pnbA demonstrates plastic polybutylene adipate terephthalate (PBAT) hydrolysis (see supplementary material), and to our knowledge is the first recorded instance of a plastic and phthalate degrading enzyme. However, further characterization of PBAT degradation is beyond the scope of this study and is a goal of future research. As a climactic study, treating PAE-polluted wastewater with thermostable and catalytically optimised mutants of pnbA would demonstrate its bioremediation utility and is also a goal in future research.

## 4 Conclusions

This study reports GAB thermophiles do contain desirable thermostable bioremedial enzymes as previously predicted by literature. This study also demonstrated that public plastic degrading enzyme databases can be used to rapidly identify phthalate degrading enzyme candidates in-silico and that phthalate/plastic degrading capabilities can be identified in overlooked non-novel enzymes. A DIBP degrading esterase called pnbA from a *Bacillus velezensis* GAB thermophile was found to have an optimal operating pH of 7–8 with stable pH activities (≥60 %) from pH's 6–8. The pnbA enzyme has an optimal operating temperature of 55 °C with moderate thermostability, retaining ≥50 % enzyme activity from temperatures 40 °C-50 °C after 7 h of heat treatment. The pnbA esterase was capable of 2-step hydrolysis of DIPB to PTH with as little as 0.122 U of pnbA able to hydrolyse 0.83 mM of DIBP within 25 min at 60 °C at a pH of 8. As a free enzyme, pnbA is better suited to be industrially produced and bioremediate DIBP than CarEW [20], due to its higher thermal stability. In future research, synthetic biology protein engineering strategies could be employed on pnbA to improve PAE degradation by creating; more thermostable mutants, fusion proteins, higher activity mutants, whole-cell biocatalysts, or a PAE-degrading superorganism to manage PAE pollution. Ideally, the engineered enzyme can be then subjected to real-world degradation scenarios like wastewater, and soil treatment.

## CRediT authorship contribution statement

**Brandon Mu:** Writing – review & editing, Writing – original draft, Visualization, Methodology, Investigation, Formal analysis, Data curation, Conceptualization. **Pawel Sadowski:** Writing – review & editing, Methodology. **Junior Te'o:** Supervision, Writing – review & editing. **Bharat Patel:** Supervision, Resources, Investigation, Conceptualization. **Nayana Pathiraja:** Writing – review & editing, Data curation. **Kevin Dudley:** Writing – review & editing, Supervision, Project administration, Funding acquisition.

## Declaration of competing interest

None.

## Data Availability

Data will be made available on request.

## Appendix Supplementary materials

Image, application 1
