# Supplementary material for: Identification and characterisation of moderately thermostable diisobutyl phthalate degrading esterase from a Great Artesian Basin Bacillus velezensis NP05
Source: Biotechnol Rep (Amst). 2024 Apr 4;42:e00840. doi: 10.1016/j.btre.2024.e00840 (PMC11033087; doi:10.1016/j.btre.2024.e00840)
Supplement: Supplementary file 1 [file mmc1.docx]

## Supplementary material

## Glossary

BCA= bicinchoninic acid assay

Da= Dalton

DIBP= Di-isobutyl phthalate

EC= Enzyme class

EDC= Endocrine disrupting hormones

GAB= Great Australian Artesian Basin

Histag= Histidine tag or poly histidine tag

HPLC= high performance liquid chromatography

IMAC=Immobilized metal affinity chromatography

IPTG= isopropyl β-d-1-thiogalactopyranoside

KDa= Kilodalton

LB= Luria broth

LMW= low molecular weight

MIBP= Mono-isobutyl phthalate

MSA= multiple sequence alignment

Ni-NTA= Nickel-nitroloacetic acid

PAE= phthalates esters

PBAT= Polybutylene adipate terephthalate

PBS= phosphate buffered saline

PMBD= Plastics Microbial Biodegradation Database

PNBE = Paranitrobenzyl esterase

pNP-C2= Paranitrophenyl ethanoate

pNP-C4= Paranitrophenyl butanoate

pNP-C6= Paranitrophenyl hexanoate

pNP-C8= Paranitrophenyl octanoate

PTH= phthalic acid

TB= Terrific broth

UB= universal buffer

PNBE accession number: OR468330

# Preliminary work methods

**Cultures and inoculum preparation:**

Five moderately thermophilic bacterial strains isolated from the Great Artesian Basin namely *Meiothermus ruber* sp. NP01, *Anoxybacillus thermarum* sp. NP02, *Anoxybacillus geothermalis* sp. NP03, *Anoxybacillus rupiensis* sp. NP04 and *Bacillus* sp. NP05 were resuscitated from stock cultures maintained at -20 ^o^C by inoculating into sterile 0.2% Trypticase Soy Broth (TSB) followed by incubation at 45 ^o^C for 2 – 3 days. All isolates were routinely cultured in the same medium at 55 ^o^C.

For biodeterioration studies, the cultures of thermophilic isolates were inoculated into 50 mL sterile falcon tubes containing 20 mL sterile MSM (NH_4_NO_3_, 1.0 g/L; MgSO_4_.7H_2_O, 0.2 g/L; K_2_HPO_4_, 1.0 g/L; CaCl_2_.2H_2_O, 0.1 g/L; KCl, 0.15 g/L; yeast extract (Difco), 0.1 g/L; and 1.0 mg/L of each of the following micro nutrients: FeSO_4_.6H_2_O, ZnSO_4_.7H_2_O and MnSO_4_ (Hadad et al.,2005)., incubated at 55 ᵒC for 36 h and their OD_600 nm_ measured. Different consortia containing equal number of cells from each isolate were prepared by mixing different volumes of the isolates. Raw sequencing data can be found at the SRA archive under project name: PRJNA1036366.

**Nylon Membrane Experimental set up:**

Polycaprolactam films (Nylon 6 with 75 micron thickness, density 1.084 g/cm^3^ _,_ [-NH(CH_2_)_5_CO-]n) purchased from Merck were cut into 15 mm x 15 mm pieces, treated with 2 % (v/v) sterile sodium dodecyl sulphate (SDS) for 4 h in an orbital shaker (200 rpm), followed by a 70% ethanol wash for 30 min and three washes with sterile deionised water each of 10 min duration, dried at 60°C overnight, weighed and stored at room temperature until used. 16 pieces of the nylon film (15 mm x 15 mm) were attached to stainless steel skewers and immersed in 48 ml Minimal Salt Medium (MSM) in 100 ml Schott bottles and sterilized. Consortia prepared as described under the previous section was used to seed the Nylon 6 film experimental setup and incubated at 55^o^C in an orbital shaker (100 rpm) for up to 30 days during which time samples were collected and analysed for evidence of biodegradation / biodeterioration. Control experiment was set up identically but without inoculation of the isolates.

**Determination of degradation products in the culture supernatants**

Aliquots (10 mL) of culture supernatants were centrifuged at 4500 rpm for 15 min and the resulting supernatant was filtered through sterile 0.2 µm filters, the eluate concentrated to dryness using a rotational vacuum concentrator (RVC 2-33IR, John Morris Scientific) at 55 °C and extracted using a fatty acid extraction kit (Catalog number MAK174, Sigma Aldrich) using a modified protocol. Briefly, 100 μL extraction solvent (Catalog number MAK174A) that contained 0.0005 mg C19 as the internal standard was added to the concentrated supernatant and mixed thoroughly for 2 min by vortex. The solvent mixture was transferred to clean 8 mL glass extraction tube with a screw cap. The extraction step was repeated with another 100 µL extraction solvent. The content in the extraction tube was dried under nitrogen for transesterification. After drying, 1 mL of 1% H2SO4 in methanol and 0.5 mL of hexane were added, capped and heated at 70 ºC for 3 hours. Then 1 mL of hexane and 1 mL of 5% NaCl were added, vortexed and centrifuged at 500 x g for 5 minutes. Top hexane layer was transferred into a glass GC vial and dried under nitrogen. Transesterified lipids were reconstituted in 100 µL of hexane and analysed using GCMS (Shimadzu 8040) with RTX 2330 column. 37 component FAME mix certified reference material (Catalog number CRM47885, Supelco) was used as the standard fatty acid mixture.

**SEM imaging of plastic films and culture supernatants**

The nylon films were washed with 1 x phosphate buffer saline (PBS), fixed overnight in 2.5% glutaraldehyde in 1 x phosphate buffered saline (PBS) followed by three washes in 1 x PBS each for 10 min. The culture supernatant (1 mL) containing bacteria from the treated nylon film were centrifuged at 10000 rpm for 15 minutes and resulting cell pellet was fixed in 2.5% glutaraldehyde in 1 x PBS overnight, washed in 1 x PBS, and attached to 10 mm round glass slides coated with poly-L-lysine. All the samples were dehydrated through a series of ethanol solutions (30%, 50%, 70%, 90%, 100%, 100%) in a Pelco Biowave. Plastic pellets were dried using hexamethyldisilazane (HMDS) and left to air dry in the fume hood. Glass slides were dried using an autosamdri-815 critical point dryer (Tousimis). Plastic films and glass slides were mounted on aluminium stubs using adhesive carbon tape (plastic pellets were also mounted using adhesive carbon paint) and coated in gold (Au) using a Leica coater. Samples were imaged at 2 kV using the in-lens secondary electron detector on a Tescan MIRA3 field emission SEM.

# Preliminary work data

### FAME-GCMS results


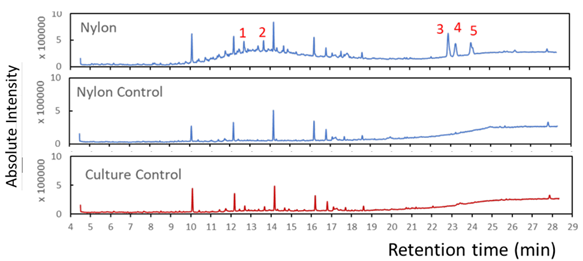


FAME-GCMS chromatograms of the degradation products in the supernatants of Nylon, incubated with thermophilic bacterial consortium, compared to plastic only controls (minimal salt medium +plastic pellets) and culture control (minimal salt medium + bacterial consortium) after 30 days of incubation at 55°C.

As shown in the figure above some unique degradation products were observed in the culture supernatants containing nylon film incubated with thermophilic bacterium consortium when compared to the controls. Based on the similarity matches to the existing libraries, the products labelled (from 1 to 5) in the figure above were identified as (1). Methyl 9- methyltetradecanoate (C16H32O2), (2). Triacontanoic acid, methyl ester (C31H62O2), (4). Thiocarbamic acid, N, N dimethyl, S-1.3-diphenyl-2- butenyl ester (C19H21NOS), and (3). and (5). as (2,3-Diphenylcyclopropyl) methyl phenyl sulfoxide, trans-(C22H20OS). Trans- (2,3-Diphenylcyclopropyl) methyl phenyl sulfoxide, and Thiocarbamic acid, N, N-dimethyl, S-1,3-diphenyl-2-butenyl ester were detected between 23 to 25 min. They were previously reported during the thermal conversion of polyethylene terephthalate (PETE) and polystyrene (PS) waste plastics into the liquid fuel materials and potentially represent derivatives of additives such as phthalates (Rashid & Sarker, 2013). Heptadecane, heneicosane, 1,54-dibromotetrapentacontane were found at 11.75, 12.44 and 13.39 min respectively in the polypropylene containing culture supernatants. Production of 1,54-dibromo tetrapentacontane, was detected during the thermal degradation of waste polypropylene into fuel oil (Sarker & Rashid, 2014). Heptadecane and heneicosane were reported during the thermocatalytic degradation of high density polyethylene (HDPE) (Patil et al., 2018).

### SEM imaging

Colonization of some bacterial strains on plastic were observed during SEM imaging as shown in the figure below. The biofilm was firmly attached and even after cleaning with a series of washes using 2% SDS, 70% ethanol and 3 x deionised water consecutively, it was observed that the biofilm was not completely removed. *Anoxybacillus rupiensis* (oval-like shape) and long rods (*Bacillus* sp.)


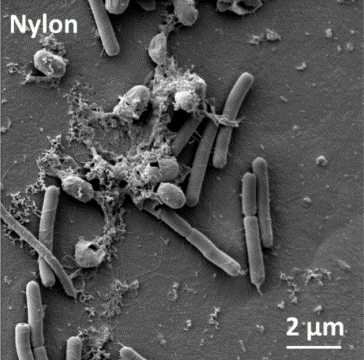


Scanning electron microscopy micrographs of the surface of nylon, after co-incubation with thermophilic bacterial consortium for 30 days at 55 °C. *Bacillus* sp. (rods), *Anoxybacillus rupiensis* (ovals)

### PMBD list

list of plastic degrading enzymes pulled from PMBD. Abbreviations; Di-isobutylphthalate (DIBP), Nylon, Polyamide (PA), polybutylene terephthalate (PBAT), Poly(butylene succinate-co-butylene adipate) (PBS), Polyethylene (PE), Polyethylene terephthalate (PET), poly(3-hydroxyalkanoate) (PHA), poly(3-hydroxybutyrate) (PHB), poly(3-hydroxyoctanoate) (PHO), phthalate, poly lactic acid (PLA), Polyurethane (PU), polyvinyl alcohol PVA, Terephthalates (TP).

| Substrate | Protein names | Organism | Gene names | Genbank ID |
| --- | --- | --- | --- | --- |
| DIBP | Carboxylic ester hydrolase | Bacillus sp. K91 | CarEW | KM098150.1 |
| DIBP | Phthalate dioxygenase large subunit | Rhodococcus sp. JDC-11 | phtAa | FJ528993.1 |
| Nylon | Endotype 6-aminohexanoat-oligomer hydrolase | Agromyces sp. KY5R | nylC | AB264778.2 |
| PA | Lipase | Pseudomonas pelagia | lip | KU695573 |
| PBAT | Aryl esterase | Pseudomonas pseudoalcaligenes | N/A | KX002004.1 |
| PBAT | Carboxylesterase | uncultured bacterium | EstB11 | KX533505 |
| PBAT | Carboxylesterase | uncultured bacterium | EstC7 | KX533507 |
| PBAT | Carboxylesterase | uncultured bacterium | EstC5 | KX533506 |
| PBAT | Carboxylesterase | uncultured bacterium | EstC9 | KX533508 |
| PBAT | Carboxylesterase | uncultured bacterium | EstG4 | KX533509 |
| PBAT | Carboxylesterase | uncultured bacterium | EstB3 | KX533504 |
| PBS | PBS(A) depolymerase | Acidovorax delafieldii BS-3 | pbsA | AB066349.1 |
| PBSA | Cutinase-like enzyme | Pseudozyma antarctica | PaCLE1 | LC276896 |
| PE | Alkane monooxygenase AlkB | Paenibacillus sp. DK1 | alkB | MK045309 |
| PEG | Polyethylene glycol dehydrogenase | Sphingopyxis macrogoltabida | pegA | AB196775.2 |
| PET | PET6 | Vibrio gazogenes | N/A | CP018835.1 |
| PET | PET12 | Polyangium brachysporum | AAW51_2473 | NZ_CP011371 |
| PET | Poly(ethylene terephthalate) hydrolase | Ideonella sakaiensis strain 201-F6 | ISF6_4831 | BBYR01000074.1 |
| PET | PET2 | uncultured bacterium | lipIAF5-2 | EU660533 |
| PET | Triacylglycerol lipase | Thermomonospora curvata | Tcur_1278 | NC_013510.1 |
| PET | Esterase | Thermobifida alba | est1 | AB445476 |
| PET | Cutinase | Thermobifida fusca | cut-2.KW3 | FR727681 |
| PET | Cutinase 1 | Thermobifida alba | cut1 | HQ147784.1 |
| PET | Cutinase 2 | Thermobifida cellulosilytica | cut2 | HQ147786.1 |
| PET | Cutinase 1 | Thermobifida fusca KW3 | cut1 | HQ147787.1 |
| PET | Esterase | Thermobifida alba | est2 | AB445476 |
| PET | LCC | uncultured bacterium | N/A | HQ704839 |
| PET | Serine hydrolase | Thermobifida halotolerans | N/A | JQ339742.1 |
| PET | PET5 | Oleispira antarctica RB-8 | lipA OLEAN_C07960 | FO203512 |
| PET | Alpha/beta hydrolase family protein | Saccharomonospora viridis | Cut190 | AB728484.1 |
| PET | Cutinase | Fusarium oxysporum f. sp. 54005 | FOQG_13916 | JH658423.1 |
| PHA | PhaZ | Pseudomonas alcaligenes | phaZ | AY370932 |
| PHA | PHA depolymerase | Paracoccus denitrificans | phaZ | AB839772 |
| PHB | Poly(3-hydroxybutyrate) depolymerase | Bacillus megaterium | phaZ | AB258388 |
| PHB | Poly(3-hydroxybutyrate) depolymerase | Talaromyces funiculosus | phaZPfu | AB281621.1 |
| PHB | PhaZd | Diaphorobacter sp. PCA039 | N/A | FJ200367 |
| PHB | PHB depolymerase | Delftia acidovorans | phaZCac | AB003186.1 |
| PHB | Poly(3-hydroxybutyrate) depolymerase | Comamonas testosteroni | phaZ | AB000508 |
| PHB | Polyhydroxybutyrate depolymerase | Pseudomonas stutzeri | phaZpst | AB012225 |
| PHB | Poly(3-hydroxybutyrate) depolymerase | Ralstonia pickettii | N/A | J04223 |
| PHB | Poly(3-hydroxybutyrate) depolymerase | Alcaligenes faecalis | PhaZ | U55775 |
| PHB | Intracellular poly(3-hydroxybutyrate) depolymerase | Cupriavidus necator H16 | phaZd | AB206256 |
| PHB | Poly(3-hydroxybutyrate) depolymerase | Ralstonia pickettii | pDS-PS | D25315.1 |
| PHB | PHB depolymerase | Schlegelella sp. KB1a | phaZ | AY538710 |
| PHB | PHB depolymerase | Azospirillum brasilense | phaZ | AF474374 |
| PHB | Poly(3-hydroxybutyrate) depolymerase | Caldimonas manganoxidans | phaZ | AB079799 |
| PHB | PHB depolymerase | Caldimonas manganoxidans | phaZ | AB038647 |
| PHB | PHB depolymerase | Acidovorax sp. TP4 | N/A | AB015309.1 |
| PHO | Poly(3-hydroxyoctanoate) depolymerase | Pseudomonas fluorescens | phaZ | U10470 |
| phthalate | Phthalate dioxygenase reductase | Burkholderia DBO1 | ophA1 | AF095748 |
| phthalate | Phthalate dihydrodiol dehydrogenase | Mycolicibacterium gilvum PYR-GCK | N/A | AY372764 |
| phthalate | Phthalate dioxygenase large subunit | Mycobacterium sp. PAH2.135 | N/A | AY372761 |
| phthalate | Phthalate dioxygenase | Burkholderia DBO1 | ophA2 | AF095748 |
| phthalate | 4,5-dihydroxyphthalate decarboxylase | Burkholderia DBO1 | ophC | AF095748 |
| phthalate | Phthalate dihydrodiol dehydrogenase | Mycobacterium sp. PAH2.135 | N/A | AY372763 |
| phthalate | Phthalate dioxygenase large subunit | Mycolicibacterium gilvum PYR-GCK | N/A | AY372762 |
| PLA | PLA depolymerase | uncultured bacterium | plaM4 | AB302136 |
| PLA | PLA depolymerase | uncultured bacterium | plaM5 | AB302137 |
| PLA | PLA depolymerase | uncultured bacterium | plaM7 | AB302138 |
| PLA | PLA depolymerase | uncultured bacterium | plaM8 | AB302139 |
| PLA | PLA depolymerase | uncultured bacterium | plaM9 | AB302140 |
| PLA | PLA depolymerase | Paenibacillus amylolyticus | PlaA | AB093482.1 |
| PLA | Cutinase-like enzyme | Cryptococcus sp. S-2 | CLE1 | AB102945.1 |
| PU | Polyurethanase A | Pseudomonas chlororaphis | pueA | EF175556 |
| PU | Polyurethanase B | Pseudomonas chlororaphis | pueB | AF188366 |
| PU | Carboxylic ester hydrolase | Delftia acidovorans | pudA | AB009606.1 |
| PU | Polyurethanase esterase A | Pseudomonas chlororaphis | pueA | AF069748 |
| PVA | Polyvinylalcohol dehydrogenase | Pseudomonas sp. VM15C | pvaA | D50670.1 |
| PVA | Polyvinylalcohol dehydrogenase | Sphingopyxis sp. 113P3 | pvadh | AB190288.4 |
| PVA | Oxidized polyvinyl alcohol hydrolase | Sphingopyxis sp. 113P3 | oph | AB190288.4 |
| PVA | Oxidized polyvinyl alcohol hydrolase | Pseudomonas sp. | pvaB | AB008494.1 |
| TP | Terephthalate 1,2-dioxygenase, reductase component 2 | Comamonas sp. E6 | tphA1II | AB238679.1 |
| TP | Terephthalate 1,2-dioxygenase, terminal oxygenase component subunit beta 2 | Comamonas sp. E6 | tphA3II | AB238679.1 |
| TP | Terephthalate 1,2-dioxygenase, terminal oxygenase component subunit alpha 2 | Comamonas sp. E6 | tphA2II | AB238679.1 |
| TP | Terephthalate 1,2-dioxygenase, reductase component 1 | Comamonas sp. E6 | tphA1I | AB238678.1 |
| TP | 1,2-dihydroxy-3,5-cyclohexadiene-1,4-dicarboxylate dehydrogenase | Comamonas sp. E6 | tphBI | AB238678.1 |
| TP | Terephthalate 1,2-dioxygenase, terminal oxygenase component subunit beta 1 | Comamonas sp. E6 | tphA3I | AB238678.1 |
| TP | Terephthalate 1,2-dioxygenase, terminal oxygenase component subunit alpha 1 | Comamonas sp. E6 | tphA2I | AB238678.1 |
| TP | 1,2-dihydroxy-3,5-cyclohexadiene-1,4-dicarboxylate dehydrogenase | Comamonas YZW-D | tphB | AY923836.1 |

# HPLC data

DIBP chromatogram 2 mM, Zorbax Eclipse XBD-c18 (5um, 4.6 x 150 mm), 30°C, 85% ACN 15% (0.1%w/v) phosphorous acid, injection volume 3 uL, Sig=230 (4nm bandwidth), ref= 360nm (100nm bandwidth), 30ml/min, 8 min run time, 2 min post time DIBP elutes at ~4.103 minutes, area= 3507.846 mAU’s


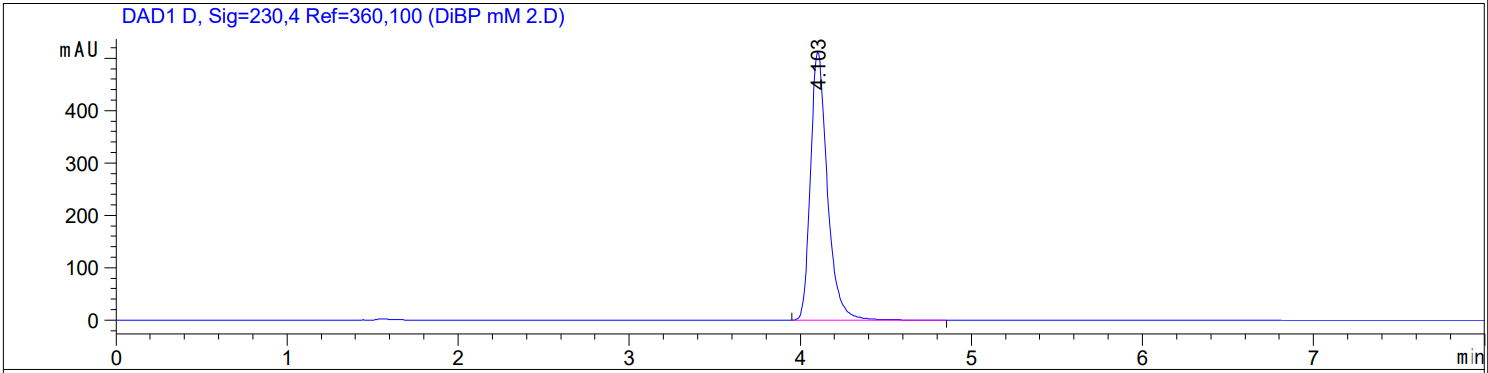


DIBP standard curve data

| DIBP mM | DIBP absorbance |
| --- | --- |
| 0 | 0 |
| 0.4 | 741.9437 |
| 0.8 | 1481.541 |
| 1.2 | 2271.862 |
| 1.6 | 2900.498 |
| 2 | 3507.846 |


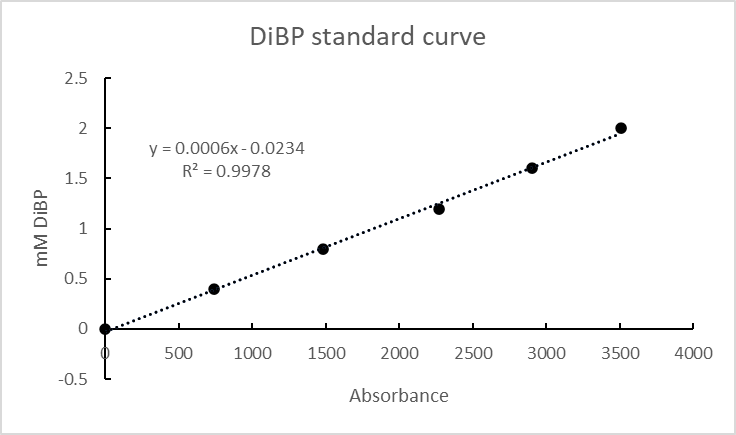


MIBP chromatogram 2 mM, Zorbax Eclipse XBD-c18 (5um, 4.6 x 150 mm), 30°C, 85% ACN 15% (0.1%w/v) phosphorous acid, injection volume 3 uL, Sig=230 (4nm bandwidth), ref= 360nm (100nm bandwidth), 30ml/min, 8 min run time, 2 min post time DIBP elutes at ~1.831 minutes, area= 2848.87305mAU’s


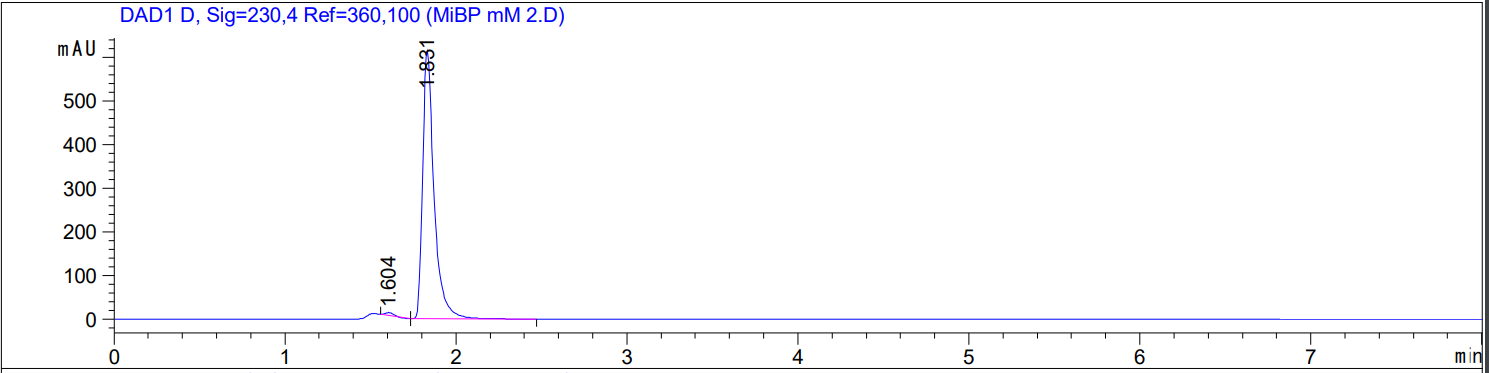


MIBP standard curve data

| MIBP mM | MIBP absorbance |
| --- | --- |
| 0 | 0 |
| 0.4 | 534.05914 |
| 0.8 | 1095.06055 |
| 1.2 | 1583.80017 |
| 1.6 | 2155.1582 |
| 2 | 2848.87305 |


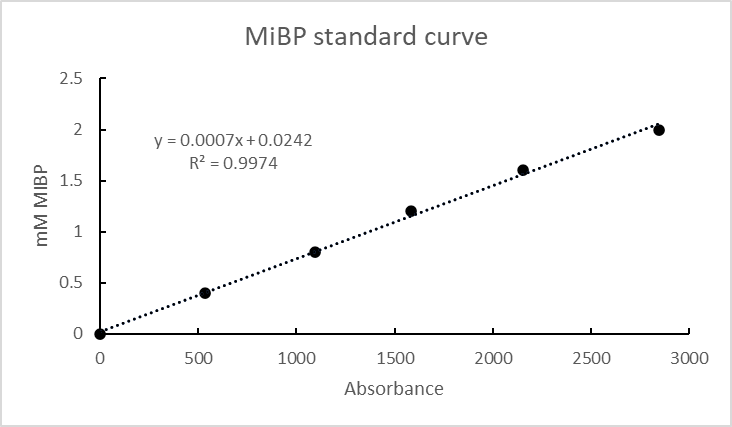


PTH chromatogram 2 mM, Zorbax Eclipse XBD-c18 (5um, 4.6 x 150 mm), 30°C, 85% ACN 15% (0.1%w/v) phosphorous acid, injection volume 3 uL, Sig=230 (4nm bandwidth), ref= 360nm (100nm bandwidth), 30ml/min, 8 min run time, 2 min post time DIBP elutes at ~1.610 minutes, area= 2587.691mAU’s


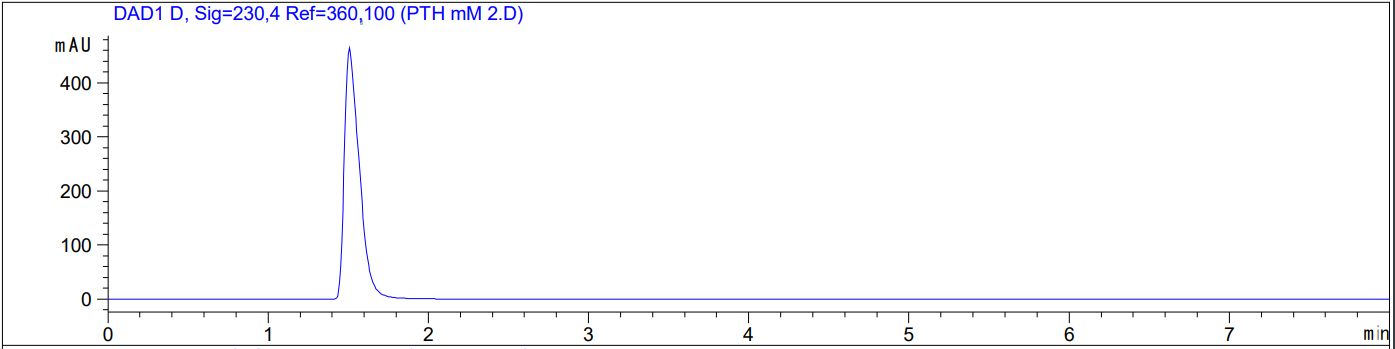


PTH standard curve data

| PTH mM | PTH absorbance |
| --- | --- |
| 0 | 0 |
| 0.4 | 581.63562 |
| 0.8 | 1064.77905 |
| 1.2 | 1639.31555 |
| 1.6 | 2427.75464 |
| 2 | 2587.69067 |

Figure below shows mystery peaks in HPLC of PNBE on DIBP, (A.) PNBE on DIBP after 25 minutes with acetonitrile co-solvent. (B.) PNBE on DIBP after 25 minutes with methanol co-solvent (mystery peak at 2.709 possibly isobutyl methyl phthalate). (C.) P PNBE on DIBP after 25 minutes with ethanol co-solvent (mystery peak at 3.098 possibly isobutyl ethyl phthalate, and mystery peak at 2.375 possibly Di-ethyl phthalate).


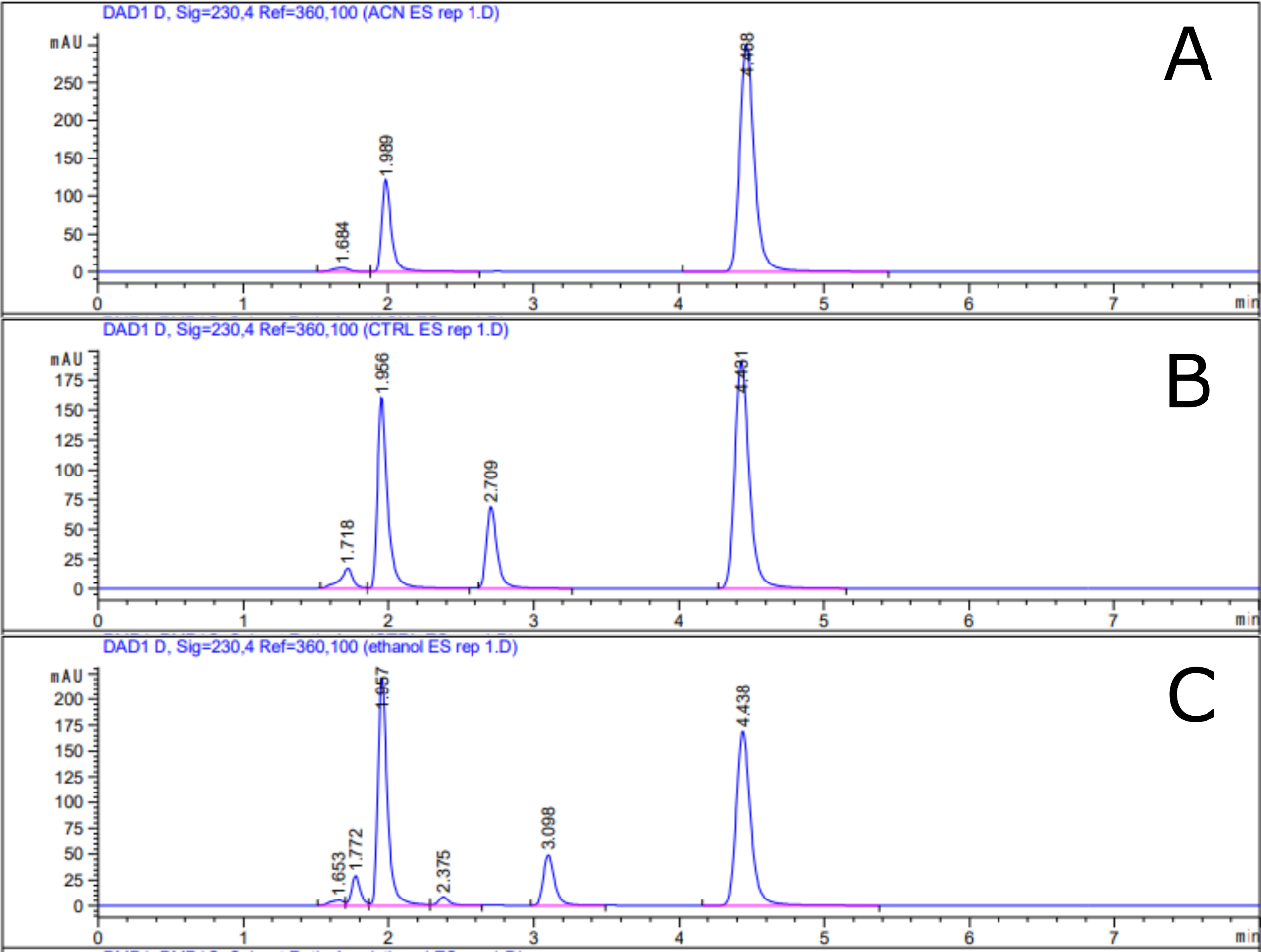


TPA chromatogram 1mM, Zorbax Eclipse XBD-c18 (5um, 4.6 x 150 mm), 30°C, 100% ACN, injection volume 5 uL, Sig=230 (4nm bandwidth), ref= 360nm (100nm bandwidth), 30ml/min, 4 min run time, 1 min post time TPA elutes at ~1.747 minutes, area= 2494.51636 mAU’s.

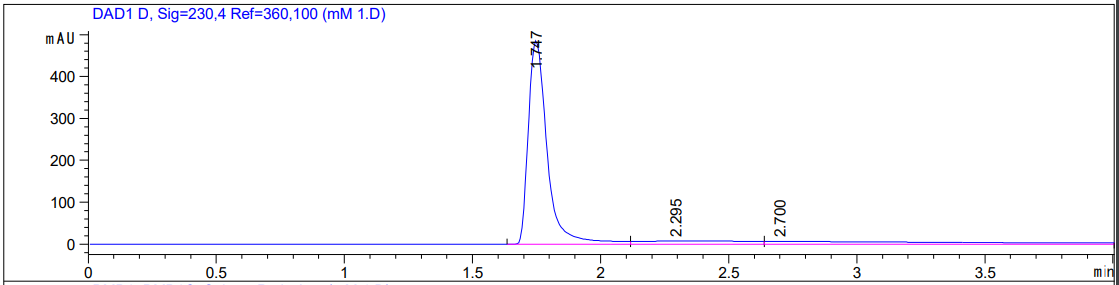


TPA std curve data

| TPA mM | TPA Absorbance |
| --- | --- |
| 0 | 179.45218 |
| 0.025 | 196.41579 |
| 0.05 | 246.07553 |
| 0.1 | 328.6051 |
| 0.2 | 607.68542 |
| 0.4 | 1036.02917 |
| 0.6 | 1542.94421 |
| 0.8 | 2113.02734 |
| 1 | 2494.51636 |


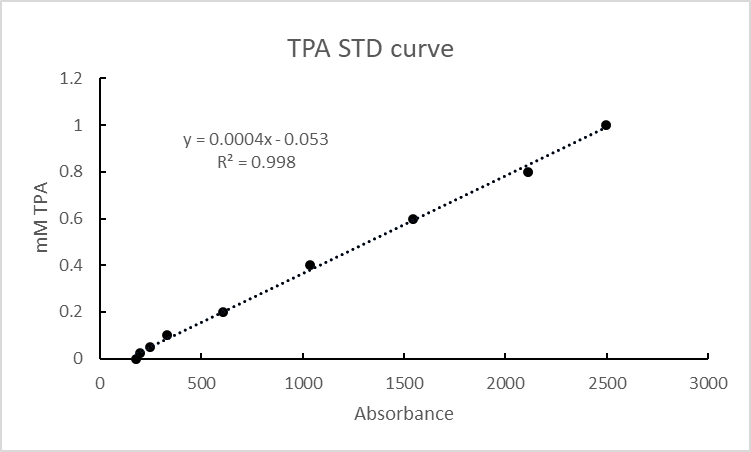


Figure below shows the degradation of polybutylene adipate terephthalate (PBAT) over 3 days by PNBE at 45°C determined by TPA quantitation. As can be seen, after 24 hours the maximal amount of TPA is produced with no increase in TPA concentration. The PBAT only controls show no change of TPA over the 3 days. This suggest PNBE is causing TPA monomer release from PBAT, likely through enzymatic degradation. The detection of TPA at days 1, 2, and 3 are significant from their respective PBAT only control groups. The TPA quantitation in enzyme only controls is noise from the HPLC.


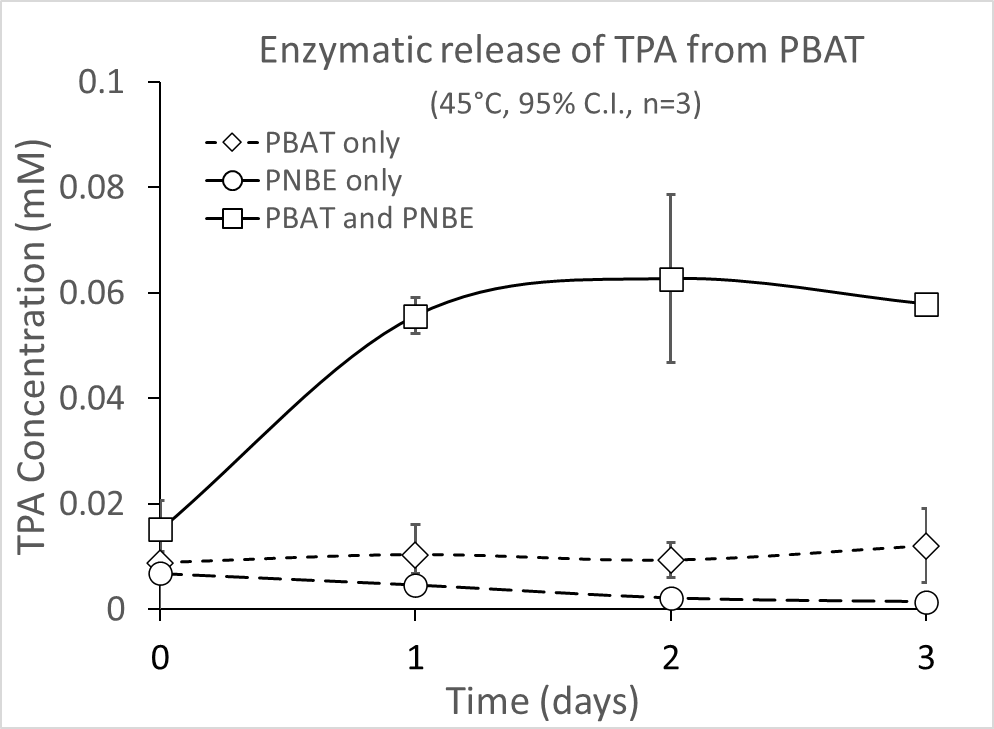


Figure 54. PNBE enzymatic degradation of PBAT to TPA monomer by TPA quantitation comparing Enzyme only ([E] PNBE only), Substrate only ([S] PBAT only), and experimental group Enzyme + Substrate ([ES] PNBE and PBAT)

HPLC data indicated PBAT enzymatic degradation by TPA quantitation. However, stronger experiment or data would confirm this giving PNBE a dual use in bioremediation (plastic and phthalate bioremedial enzyme).
